# Intended and Unintended Benefits of Folic Acid Fortification—A Narrative Review

**DOI:** 10.3390/foods12081612

**Published:** 2023-04-11

**Authors:** Shrooq Ismail, Sereen Eljazzar, Vijay Ganji

**Affiliations:** Human Nutrition Department, College of Health Science, QU Health, Qatar University, Doha P.O. Box 2713, Qatar

**Keywords:** anemia, folic acid, folic acid fortification, homocysteine, cardiovascular diseases, USA

## Abstract

Inadequate folate intake during pregnancy is the leading cause of the development of neural tube defects (NTDs) in newborns. For this reason, mandatory fortification of folic acid, a synthetic, easily bioavailable form, in processed cereals and cereal products has been implemented in the US since 1 January 1998 to reduce the risk of NTD in newborn children. This report aimed to review the literature related to the impact of mandated folic acid fortification on the intended and unintended benefits to health. Potential adverse effects were also discussed. We searched Pubmed, Google Scholar, Embase, SCOPUS, and Cochrane databases for reports. About 60 reports published between January 1998 and December 2022 were reviewed, summarized, and served as background for this review. The intended benefit was decreased prevalence of NTDs, while unintended benefits were reduction in anemia, blood serum homocysteine, and the risk of developing cardiovascular diseases. Potential issues with folic acid fortification are the presence of unmetabolized folic acid in circulation, increased risk of cancer, and the masking of vitamin B-12 deficiency. From a health perspective, it is important to monitor the impact of folic acid fortification periodically.

## 1. Introduction

Folate (vitamin B9) is a water-soluble vitamin that is found in many foods. Folate can exist in one of two forms, either the reduced, naturally occurring folate or the oxidized, synthetic folic acid. Folic acid is used in supplements and to fortify cereals and processed grain products [1]. Dietary sources of folate include green leafy vegetables, legumes, beans, and organ meats. The bioavailability of folic acid is much higher than natural folate because it is present in the monoglutamate form, while folate requires the enzyme conjugase for its digestion [2]. Folic acid is also more stable in oxidation and heat compared to natural folate [2]. In the past, efforts were made to use methyl tetrahydrofolate (MTHF) as an alternative to folic acid as a fortificant because it is a natural and biologically active form with fewer potential health risks. MTHF utility is limited because it is less stable than folic acid in foods that undergo thermal processing. However, more research is needed to determine the efficacy and safety of MTHF as a fortificant [3,4]. 

Folic acid fortification has been implemented in many countries due to the widespread dietary inadequacy of this vitamin, especially in women of childbearing age [5]. The main objective of folic acid fortification was to prevent neural tube defects (NTDs) in newborns [5]. Since the implementation of folic acid fortification, it was estimated that 18% of all potential folic acid-preventable NTDs were prevented worldwide in 2017 and 22% were prevented worldwide in 2019 [6].

Globally, not every country embraced the mandated folic acid fortification policy. For example, there are significant differences in folic acid fortification policies between the US and Europe. In Europe, mandatory fortification of food with folic acid is not widespread. Some European countries such as the UK, have voluntary folic acid fortification policies, while others have no fortification policy at all. The debate on whether to introduce mandatory folic acid fortification across Europe is still ongoing. Some concerns are the long-term safety of folic acid, the potential masking of vitamin B-12 deficiency, and a possible role in cancer are still being discussed [7,8].

In addition to its intended benefit of preventing NTDs, folic acid fortification brought some unintended benefits. These were a reduction in anemia (improved hemoglobin concentrations), decreased risk of cardiovascular diseases (CVD), and improved cognitive function [9]. A few reviews have been published on the health effects of folic acid supplementation. This is the first comprehensive review of the intended and unintended benefits of mandated folic acid fortification legislation on public health nutrition. It has been 25 years since the folic acid fortification legislation has been implemented in the US. We have reviewed the literature and synthesized the evidence on the health effects of mandated folic acid fortification using the studies reported after 1 Jan 1998. Therefore, the purpose of this overarching review was to assess the intended and unintended benefits of mandated folic acid fortification of processed cereals and cereal products in the US.

### 1.1. Biochemical Role of Folic Acid/Folate

**Figure 1:**
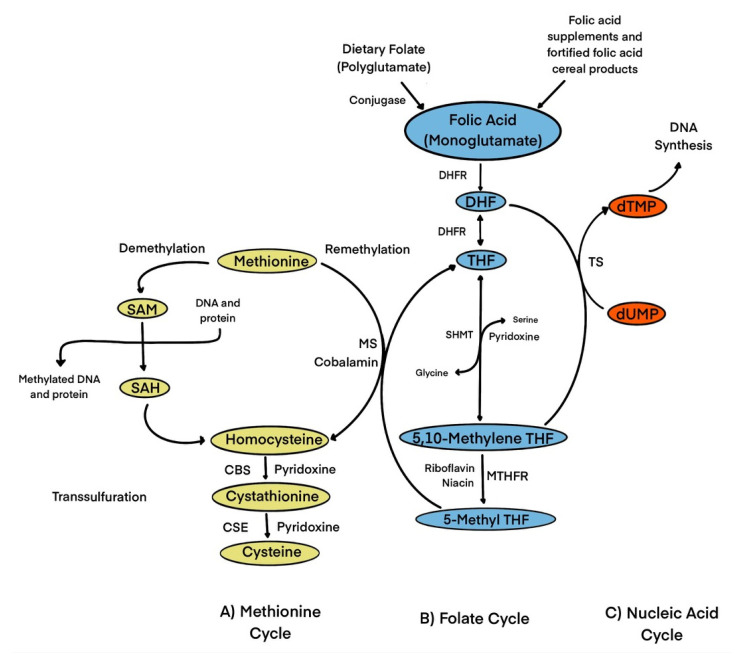
Role of folate in one-carbon transfer metabolism. Folic acid from dietary folate or folic acid from fortified foods/supplements is reduced to DHF and then to THF by DHFR. SHMT converts THF to 5,10-methylene THF. 5,10-methylene-THF is reduced by MTHFR to 5-methyl-THF. 5-methyl THF is catalyzed by MS to generate THF and methionine through the remethylation of Hcy. Cobalamin is a cofactor for MS. Methionine is then used to form SAM, which serves as a universal methyl donor for numerous reactions and produces SAH, which then generates homocysteine. Hcy is then used either to regenerate methionine, or it is converted to β-cystathionine and then to cysteine in the transsulfuration pathway. In the regeneration of DHF from 5,10-methylene THF, dUMP is converted to dTMP by TS which can be used for DNA synthesis. Abbreviations: CBS, cystathionine-β synthase; CSE, Cystahionine-γ lyase; DHF, dihydrofolate; DHFR, dihydrofolate reductase; dTMP, deoxythymidine monophosphate; dUMP, deoxyuridine monophosphate; MS, methionine synthase; MTHFR, methylenetetrahydrofolate reductase; SAH, S-adenosylhomocysteine; SAM, S-adenosylmethionine; SHMT, serine hydroxymethyltransferase; THF, tetrahydrofolate; TS, thymidylate synthase.

Folic acid acts as a precursor, cofactor, and substrate for various biological processes such as nucleotide synthesis through one-carbon transfer pathways [1]. These pathways utilize nutrients such as glucose, vitamins, and minerals to fuel many metabolic processes [10]. These biochemical pathways utilize folic acid and methionine to produce methyl groups that are essential for processes such as DNA synthesis, antioxidant generation, and epigenetic regulation [1]. The production of S-adenosylmethionine, the universal methyl donor for these methylation relations, is a crucial part and is what mediates these reactions, which is a critical step in mediating these processes [10]. Figure 1 shows a brief overview of the metabolism of folic acid [11].

### 1.2. Dietary Reference Intakes (DRI), Adequate Intakes (AI), and Tolerable Upper Intake Levels (UL) of Folate/Folic Acid

DRI is the average daily level of intake that is needed to meet the nutritional requirements of almost all healthy individuals. In the absence of DRI, an AI can be estimated to ensure nutritional adequacy [8]. The US Food and Nutrition Board defines these recommendations in terms of Dietary Folate Equivalents (DFEs) to reflect the fact that folic acid is more bioavailable than natural food folate (85% bioavailability for the former compared to 50% for the latter. Therefore, 1 μg of DFE can either be 1 μg of folate from food, 0.6 μg folic acid from supplements or fortified food, or 0.5 μg of folic acid taken on an empty stomach [8]. The DRI for adults aged ≥19 years old is 400 µg of DFE for men and women. During pregnancy and lactation, the DRI is 600 and 500 µg DFE, respectively [8]. Folic acid also has a UL which indicates the maximum daily intake that would be unlikely affect health. The UL for adult men, women, and pregnant and lactating women over the age of 18 years old is 1000 µg. No UL was given for food folate [8].

### 1.3. Biomarkers of Folic Acid Status

There have been four biomarkers proposed to estimate folate status. These are plasma or serum folate, red blood cell (RBC) folate, mean corpuscular volume (MCV) concentrations, and plasma total homocysteine (tHcy) [9]. A folate deficiency is defined as a serum folate concentration of <7 nmol/L or an RBC folate concentration of <312 nmol/L [8]. A folic acid deficiency causes megaloblastic anemia or macrocytosis. Many factors may contribute to the deficiency of folic acid, including states of increased energy requirements such as pregnancy, lactation, alcoholism, and losses during food preparation, as well as the use of certain medications [2]. 

Today’s Westernized diets contribute largely to the unmet folic acid requirements [12]. The replacement of complex carbohydrates and vegetables with refined sugars and carbohydrates, as well as the absence of whole foods, are the causes of widespread folic acid deficiency. Fad diets such as low-carbohydrate or ketogenic diets may also contribute to folate deficiency due to the decreased intake of commonly fortified foods, such as cereals and bread [12]. 

### 1.4. Folic Acid Fortification

To date, more than half of women of childbearing age fail to meet the folic acid requirement for preventing NTDs [13]. Therefore, the US Public Health Service recommends that women of childbearing age consume 400 μg of folic acid daily for NTD prevention, but no more than 1000 μg/day because of the unknown health effects of excessive folic acid. This can be achieved through diet alone, particularly in countries with mandated folic acid fortification legislation. Supplementation with 400 μg of folic acid per day is also recommended for the prevention of NTDs [13].

The USA was the first nation to implement mandatory folic acid fortification for the prevention of NTDs, which has significantly reduced their incidence [14]. Currently, more than 80 other countries have adopted the US policy of fortifying cereal grains with folic acid [14]. Fortified products include staple items such as foods like bread, four, rice, pasta, and breakfast cereals [15]. Folic acid fortification programs aimed to decrease NTD incidence among women of childbearing age; however, because folic acid fortification is not selective and applies to the entire population, other subgroups such as men, children, and elderly persons have seen a rise in serum folate concentrations. This increase has resulted in unintended benefits alongside the intended benefit of decreasing NTDs. Unfortunately, some potential health issues have been reported regarding excessive folic acid consumption, but more research is needed to confirm these reports [8].

## 2. Methods

For this narrative review, we obtained reports by searching electronic databases such as PubMed, EMBASE, SCOPUS, and Google Scholar. We also conducted an additional free-hand search to ensure that this review includes relevant papers and to reduce the possibility of missing any relevant papers. Our inclusion criteria were reports published between 1998 and 2022. We used main keywords such as ‘folic acid’, ‘folate’, ‘fortification’, ‘neural tube defects’, ‘NTDs’, ‘United States’, and ‘U.S.’ to identify relevant studies. We included only those reports that were based on the US population. Only those studies that were reported in English were considered in this review. Two reviewers independently screened all the articles by publication titles and abstracts and then assessed and evaluated the full text for inclusion. Any disagreements were resolved through a second review and discussion among the authors.

## 3. Results and Discussion

### 3.1. Intended Benefits of Folic Acid Fortification

NTDs are a form of birth defects that results from the failure to close the neural tube enclosure in the early gestational period. NTDs can be classified as “open” or “closed” depending on whether the neural tissue is covered. This defect can cause anencephaly, spina bifida, as well as some other congenital defects and abnormalities [16]. The prevalence of fatality cases due to spina bifida is ≈7% compared to other NTDs, i.e., encephalocele (46%) and anencephaly (100%) [17]. However, this difference may be due to a higher pregnancy termination rate after the prenatal diagnosis of spina bifida [17].

Folic acid status contributes to NTDs through several suggested mechanisms and hypotheses. Some researchers have suggested that the presence of elevated folate receptor antibodies would limit folate transport in the early stages of embryo development, leading to developmental impairment [18]. Others have hypothesized that folate has a role in DNA methylation, which results in the over-expression of some genes involved in autoimmunity associated with the progression of NTDs [18]. Studies have observed a dose-dependent effect between serum folic acid and the risk of NTD incidence. The lower the serum folic acid, the higher the risk. This explains the higher beneficial effects in countries with higher NTD prevalence following folic acid fortification [18]. Genetics, race-ethnicity, sex-related epigenetic changes, maternal age, and socioeconomic status play a role in the development of NTDs, but folic acid also serves as an independent environmental predictor in the pathogenesis of these diseases [19].

Since 1965, the relationship between folic acid and NTDs has been well established, and the combination of supplementation and fortification of folic acid has been successful in fulfilling their main goal of preventing various NTDs. Due to mandatory food fortification programs, the incidence of NTDs has decreased by up to 22% of cases of folic acid-preventable NTDs in 2019 alone [20,21].

The cost-effectiveness of folic acid fortification has been studied in several projects. One study conducted in the US investigated the cost-effectiveness of folic acid fortification and showed that folic acid fortification decreased the present value of total direct costs for each year of birth by $603 million more than the cost of fortification. Additionally, the number of infants born with spina bifida was reduced by 767 each year [21]. The first-year survival rate among 2841 infants with spina bifida and 638 with encephalocele, who were born between 1995 and 2001, was also studied [22]. The survival rate for infants with spina bifida during the mandatory fortification period was 92.1% (adjusted HR: 0.68; 95%CI: 0.5, 0.91), an improvement compared to the 90.3% survival rate before fortification. However, no significant improvement was observed in encephalocele for the first-year survival rate. This evidence shows that folic acid fortification plays a significant role not only in reducing the prevalence of NTDs but also improving the severity of spina bifida and first-year survival rate [22].

Post-fortification, it has been shown that the highest reduction in NTDs was observed among the Hispanic population, who initially had a higher prevalence of NTDs compared to the African American population within the US [23]. Another study showed that although the rate of spina bifida in the Hispanic population declined with time, it remained constantly higher when compared to white or black populations [24]. This has been explained in part by the higher mutation rate of the C677T methylenetetrahydrofolate reductase (MTHFR) homozygous genotype [24].

Other possible reasons for the reduction in NTD cases include medical and surgical interventions, which have improved with advancements in intensive care support and the use of antibiotics to treat central nervous system infections, leading to better treatment of NTDs [23]. Additionally, there have been improvements in associated comorbidities, such as low birth weight and prematurity, which have been identified as strong factors for infant deaths [23]. Improvements in surgical interventions for spina bifida and other related defects, such as hydrocephalus and cardiac defects, may also contribute to the reduction in NTD-related death rates, which are unrelated to folic acid fortification [18].

Numerous studies have evaluated optimal serum folate concentrations to reduce the risk of NTDs. The World Health Organization recommends maintaining a serum folate concentration of at least 906 nmol/L to decrease this risk [25]. Folic acid supplements are usually recommended in pre-pregnancy due to the greater amounts of folic acid needed for the rapid rate of cellular and tissue growth during early pregnancy [25]. Table 1 presents studies with the intended and unintended benefits of folic acid.

Folate metabolism may differ depending on pregnancy status. One study compared two diets consisting of two levels of folic acid (450 μg from food vs. 850 μg from folic acid fortified foods) given to pregnant and non-pregnant women. This study showed that pregnant women compared to non-pregnant women are more efficient at conserving folate, especially when given at a lower dose [25]. Although urinary excretion is increased at both doses, this suggests that both lower and higher levels may maintain the necessary folic acid concentrations [25].

### 3.2. Unintended Benefits of Folic Acid Fortification

#### 3.2.1. Reduction in tHcy

Worldwide, CVDs are among the most prominent global public health challenges of the 21st century [34]. Many studies have established that homocysteine (Hcy) is considered an independent risk factor for CVD. It has been reported that more than 60% of cases with CVD were also simultaneously diagnosed with hyperhomocysteinemia, although further research is needed regarding its management and treatment [34]. Hcy is a non-essential, sulfur-containing amino acid that is produced by methionine through a demethylation reaction [34]. In the human body, 50% of the Hcy is remethylated to form methionine. This reaction requires several enzymes as well as three vitamins that serve as coenzymes, including folic acid, vitamin B-12, and vitamin B-6 [34]. In the conversion of Hcy into methionine, folate in the form of MTHF provides Hcy with a methyl group in the remethylation catalyzed in the presence of vitamin B-12 by methionine synthase [35].

Elevations in plasma tHcy are multifactorial and may be affected by factors such as genetics, enzyme dysfunction, and vitamin deficiencies [35,36]. A deficiency in pyridoxine and cyanocobalamin coenzymes can lead to an increase in total plasma tHcy concentrations [37]. Genetic defects in enzymes required in the remethylation pathway that may lead to hyperhomocysteinemia include MTHFR, methionine synthase, and the first step of the transsulfuration pathway responsible for cystathionine β-synthase. Furthermore, lifestyle factors such as smoking, coffee, and alcohol consumption, and some medical conditions such as diabetes and renal impairment, may contribute to elevated plasma tHcy concentrations [37]. 

Circulating tHcy concentrations can be assessed by measuring serum MTHFR concentrations [34]. Elevation of circulating tHcy concentrations may cause organ dysfunction, leading to an increase in the risk of venous thromboembolism, arterial thrombosis, and early development of CVDs [36]. Some studies showed that high concentrations of tHcy in the blood, along with a folate deficiency, are significantly associated with the risk of myocardial infarction [38]. Other studies have linked plasma tHcy and the risk of fractures due to osteoporosis in the elderly [38].

One unintended benefit of increasing folic acid in diets through folic acid fortification has been a reduction in circulating tHcy concentrations. The association between tHcy and folic acid fortification was previously studied in 1999 in a cohort study [36]. The authors determined the impact of folic acid fortification on serum folate and tHcy concentrations. They compared individuals exposed to folic acid fortification (between the years 1997–1998) to those who were not (between the years 1995–1996) [36]. They found that those exposed to folic acid fortification had a significant increase in mean folic acid concentrations, as well as a reduction in tHcy compared to baseline concentrations, while the control group had no significant reductions in tHcy [36].

A retrospective study using National Health and Nutrition Examination Surveys (NHANES) 1988–2002 reported on the temporal association of folic acid fortification and serum folic acid, RBC folate, and tHcy concentrations [26]. This study showed a reduction in serum folate with a mean concentration of 149.6% in 1999–2000 and 129.8% in 2001–2002, both significantly higher than in 1988–1994 even after adjusting for age, sex, and race/ethnicity. Hcy was also reduced from 9.5 μmol/L in 1988–1994 to 7.6 μmol /L in 1999–2000 and to 7.9 μmol/L in 2001–2002 after adjusting for various confounders. The reduction in serum folate in 2001–2002 compared to 1999–2000 was justified predominantly in people with high folate intake levels, rather than in those with lower intake levels [26]. This shows that folic acid fortification contributed to significant improvements in folate status. However, over time, serum folate concentrations slightly declined in 2001–2002 due to lower folic acid intake [26].

One meta-analysis published in 2014 observed the difference in tHcy reduction among groups with fortification, partial fortification, and without fortification [39]. This study found that the tHcy reductions were 27%, 18.4%, and 21.3% in the subgroups without folate fortification, with folate fortification, and with partial folate fortification, respectively. During the same period, the reduction in stroke mortality decreased slightly in Canada and the US after folate fortification [39]. The causes and effects between folic acid fortification and decreased risk of CVD are difficult to demonstrate because most trials had participants who already had a high risk of CVD and may have already received intensive lipid profile interventions before folic acid fortification. Thus, the impact of tHcy on lowering the risk of CVD is difficult to demonstrate in these trials. On the other hand, in populations not taking lipid-lowering medication with a middle to high risk of cerebrovascular events, the effect of folate might be more evident [39].

Similar results have been found in another meta-analysis including 24 randomized control trials (RCT) examining the relationship between folic acid supplementation and glycemic control [40]. The results showed significant reductions in fasting insulin [weighted mean difference (WMD): −1.63 pmol/L; 95%CI: −2.53, −0.73], fasting blood glucose (WMD: −2.17 mg/dL; 95%CI: −3.69, −0.65), and HOMA-IR (WMD: −0.4; 95%CI: −0.7, −0.09) following folic acid supplementation. However, the effect on HbA1c was not significant. This concludes that folic acid supplementation may reduce blood glucose-related biomarkers [40]. However, the reductions were minimal and may limit their clinical applications for adults with diabetes. Unfortunately, most of the studies have been conducted in regions without mandatory folic acid fortification and focused on studying the effect of folic acid supplementation [40] (Table 1).

#### 3.2.2. Impact on Stroke and CVD

Folic acid fortification has been shown to decrease tHcy concentration, and folic acid supplementation is a debatable option for reducing tHcy concentration. Research has shown that 0.5–5 mg of folic acid can reduce tHcy by 25%, which can lower the risk of CVDs. It has been established that lowering tHcy concentrations by 3–4 µmol/L reduces the risk of CVD by 30–40% [36,37]. One meta-analysis of 12 RCTs showed that participants who received folic acid supplementation had a significantly decreased risk of stroke compared to those who did not (RR: 0.85; 95%CI: 0.77, 0.94) [34]. However, there were no significant differences in the other outcomes such as CVD mortality, all-cause mortality, and risk of coronary heart disease (CHD) [41]. Consistent with their findings, another meta-analysis was carried out on 11 RCTs, including 65,790 individuals with CVD, and found that stroke incidence in patients with pre-existing CVD was significantly reduced (RR: 0.9; 95%CI: 0.84, 0.97; *p* = 0.005) [42].

Several studies have investigated the mechanism behind lowering the concentration of tHcy by giving folic acid in cases of the MTHFR 677C→T genotype. One meta-analysis showed that cases with the polymorphisms in MTHFR 677TT had a 16% (OR: 1.16; 95%CI: 1.05, 1.28) higher risk of chronic heart disease compared with individuals with the CC genotype [43]. However, after dividing the genotype groups into high or low folic acid status, the genotype effects were noticed to be invalid in the high folic acid status group [43]. There was a similar risk for CHD in the MTHFR 677TT genotype adults seen in patients with the CC genotype (OR: 0.99; 95%CI: 0.77, 1.29). Cases with the MTHFR 677TT genotype who had low folic acid concentrations were at higher risk for CHD (OR: 1.44; 95%CI: 1.12, 1.83) compared to those with high folic acid status and the CC genotype [43]. Although all the included studies were for folic acid supplementation, the phenomena of impacts of the higher risk genotype are overcome by adequate serum folic acid concentrations which can be reached by folic acid fortification [43]. 

Similar results were reported in a study that was conducted to investigate folate’s role in the association between MTHFR 677C→T and stroke in the genetic analyses [27]. A meta-analysis of 13 randomized trials concluded that the effect of the MTHFR 677C→T variant on tHcy concentration in low folate concentration regions such as Asia (difference between cases of TT vs. CC genotype: 3.12 μmol/L; 95%CI: 2.23, 4.01) was higher in comparison to areas with folate fortification such as the US (high: 0.13 μmol/L; 95%CI: −0.85, 1.11) [27]. In addition, the odds ratio for stroke was higher in Asia (OR: 1.68; 95%CI: 1.44, 1.97) than in the US, Australia, and New Zealand, (OR: 1.03; 95%CI: 0.84, 1.25) [27].

A population-based cohort study was conducted to compare the stroke mortality rates in Canada and the US between 1990 to 2002, during the period of fortification, with the mortality rates in England and Wales, which did not adopt the folic acid fortification policy [28]. This study found that stroke mortality decreased by an average of −1.9% (95%CI: −1.4, −0.6) per year from 1990 to 1997 and −5.4% (95%CI: −6, −4.7) per year from 1998 to 2002 (*p* ≤ 0.0001) [28]. However, the effect of folic acid fortification on tHcy concentration and CVD remains controversial due to several factors such as participants’ health condition, the presence of kidney disease, the dose of folic acid, and interaction with other vitamins such as vitamin B-12 or vitamin B-6. More studies are needed to clarify this topic.

#### 3.2.3. Impact on Cognitive Health and Depression 

The relationship between folic acid and cognitive performance in adults is still not fully understood. The major role of folate during fetal development has been studied and linked to neuronal structure and function, cell polarity and plasticity, and vesicular transportation [44]. A study on folic acid fortification assessed the effect of folic acid fortification from food alone on the intelligence quotient (IQ) in children aged 6 years old with mothers diagnosed with epilepsy and on anti-seizure medication (ASM) [45]. They found that folic acid from food alone was not associated with the enhancement of IQ in children aged 6 years old. In contrast, those children who took folic acid supplements found a 10.1-point higher increment in IQ levels (95%CI: 5.2, 15; *p* < 0.001) [45]. This shows that dietary folic acid from fortification, even in a country where food is fortified with folic acid, is not adequate to improve cognitive outcomes for children of women using ASMs during pregnancy [45].

Another study conducted among 292 youths aged 8 to 18 years old studied the associations among fetal folic acid exposure, cortical maturation, and psychiatric risk [46]. This study showed exposure-associated cortical thickness increases in bilateral frontal and temporal regions (9.9% to 11.6%; *p* < 0.001 to *p* = 0.03) and the emergence of quadratic age-associated thinning in temporal and parietal regions (β = −11.1 to −13.9; *p* = 0.002) and flatter thinning profiles in frontal, parietal, and temporal areas were associated with inferior odds of psychosis spectrum symptoms [46].

One cohort study assessed folic acid concentrations and their relationship with cognitive function and dementia in Latinos aged >60 years old who were exposed to folic acid fortification. After assessing cognitive health with a Modified Mini-Mental State Examination (3MSE), and a cross-culturally validated neuropsychological test, they found that the concentration of RBC folate was directly associated with cognitive function scores and inversely associated with dementia, despite folic acid fortification [47]. Additionally, RBC folate concentration was associated with 3MSE and delayed recall scores after adjusting for several confounding factors. Furthermore, the relative risk of cognitive impairment and dementia decreased with increasing RBC folate concentration [47]. In contrast, a cohort study using the NHANES data from 1999–2002 in elderly individuals above 60 years old found a direct association between high serum folate and both anemia and cognitive impairment in those who had low serum vitamin B-12 concentration during the age of folic acid fortification. On the other hand, a combination of normal vitamin B-12 status and high serum folate was found to be protective against cognitive impairment [29].

Similar results have been found in another meta-analysis, which indicated a dose-response relationship between folic acid and the risk of cognitive impairment in older adults with vitamin B-12 deficiency. The study suggested a “J shape” relationship between serum folate concentration and cognitive impairment [48]. The suggested mechanism behind this relationship is that elevated folate might mask vitamin B-12 deficiency. Epidemiological studies have shown that individuals with low serum vitamin B-12 in combination with high folate present a higher risk of cognitive impairment compared to those with normal serum folate, especially in the elderly population due to lower absorption of vitamin B-12 [29,48]. It has been estimated that folic acid fortification might be associated with an increase in the risk of cognitive impairment in up to 4% of older adults in the US [29]. Current regulations and fortification policies should be addressed to analyze the risk-benefit to weigh public health risks and benefits.

#### 3.2.4. Depression

About 8% of the US population has depression [49]. Folate plays an important role in the nervous system functioning through one-carbon transfer reactions. Although no study has directly linked folic acid fortification with decreased depression, a few studies investigated the relationship between folic acid supplementation and depression. Subjects with depression had low folate status and elevated tHcy leading to a reduced supply of methyl groups. This further leads to decreased methylation of monoamine neurotransmitters such as serotonin, dopamine, and nor-epinephrine in the brain, which have been implicated in mood disorders [50]. A study found that folate deficiency was observed in up to a third of individuals with severe depression. A collective analysis of six RCTs demonstrated that supplementation of folic acid resulted in a decrease in depression scores, as measured by the Beck Depression Inventory (WMD: −3.9; 95%CI: −5.3, −2.4). Additionally, it resulted in a lower depression score on the Hamilton Depression Inventory (WMD: −3.5; 95%CI: −4.6, −2.4); *p* < 0.001) compared to the control group [51]. A meta-analysis consisting of data from 15,315 participants (1769 subjects with depression and 13,546 control subjects) revealed a significant association between folate status and depression (pooled adjusted OR:1.42; 95%CI: 1.1, 1.83) [52]. Therefore, it is recommended that individuals with depression or at risk of depression ensure that they are getting adequate amounts of folate through diet or supplements. 

#### 3.2.5. Reduction in Anemia 

Anemia is a major public health concern in both developing and developed countries, caused by several micronutrient deficiencies, including ferrous sulfate, vitamin B-12, and folate [53]. Iron deficiency anemia is the most common form, accounting for over half of all anemia cases. Viral infections and inflammation are also the causes of anemia [53]. The prevalence of anemia in developing countries is almost 43%, compared to 9% in developed countries [53]. Anemia has many adverse effects on health, including compromising immunity, reducing cognitive performance, and lowering productivity. Moreover, severe anemia is a major risk factor for maternal and infant morbidity and mortality [54]. Folate deficiency is typically associated with macrocytic anemia, which can cause megaloblastic changes in the bone marrow and macrocytosis in red blood cells [54].

In an observational study that compared the prevalence of anemia in children before and after double fortification of wheat flour with folic acid and iron, no significant difference was found in mean hemoglobin concentrations between pre-fortification and post-fortification. However, MCV concentrations increased significantly in the post-fortification period from the pre-fortification period (from 76.8 fL to 79.1 fL; *p* = 0.02) [30]. Another study used NHANES data to evaluate the prevalence of anemia and macrocytosis before and after folic acid fortification (1988–1994 vs. 1999–2004) [31]. The study found a significant increase in hemoglobin from 1988–1994 to 1999–2004 with a rise from 15.1 to 15.4 mg/dL (*p* < 0.0001) in men and 13.3 to 13.6 g/dL (*p* < 0.0001) in women [31]. MCV status also improved during this time frame, with significant increments observed in men (from 90.2 to 90.7 fL; *p* = 0.0123), in senior men ˃60 years (from 91.6 to 92.4 fL; *p* = 0.0105), and in women (from 90.7 to 91.4 fL; *p* = 0.0141). The prevalence of anemia was only significant in women, with a 27.9% reduction (*p* = 0.0005) in 1999–2004 compared to 1988–1994. Furthermore, the odds of having anemia in post-fortification compared to pre-fortification were 0.64 in women (95%CI: 0.54, 0.75; *p* < 0.0001) and 0.79 in men (95%CI: 0.62, 0,99; *p* < 0.0433). However, no significant effects were observed on the odds of macrocytosis after folic acid fortification [31].

In contrast, Hirsch et al. found an increase in MCV in the post-fortification period compared to the pre-fortification period, suggesting an increase in vitamin B-12 deficiency [55]. The study suggested that higher doses of folic acid supplements (more than 1000 μg) could mask hematologic symptoms associated with vitamin B-12 deficiency. This could lead to a missed diagnosis of vitamin B-12 deficiency as irreversible neurological impairment may occur even in the presence of normal hematology [55]. 

One RCT found that six months after the fortification of maize flour, women who consumed the fortified flour had a significant increase in hemoglobin concentrations (mean 13.3 g/dL compared to 13.1 g/dL at baseline) [32]. However, a study carried out in China found no significant effects in the prevalence of anemia among women who consumed folic acid-fortified wheat flour compared to the control group after 36 months of intervention (RR: 0.87; 95%CI: 0.68, 1.11) [56].

The effect of folic acid fortification remains controversial due to several reasons, such as the target population, the amount of folic acid fortification, the cut-off point of hemoglobin, and whether the difference in hemoglobin is clinically significant. Moreover, the heterogeneity and residual confounders of studies are limitations. Further research is needed to explore the effect of folic acid fortification on populations with anemia, especially macrocytic anemia.

#### 3.2.6. Diabetes

Diabetes can cause some complications related to macro- and micronutrients. The implication of folic acid in the pathogenesis of type 2 diabetes (T2DM) is associated with vitamin D deficiency and its consequence in hyperhomocysteinemia [57]. A case-control study found that low intakes of folate and vitamin B-12 in T2DM were associated with hyperhomocysteinemia and DNA damage, as measured by the presence of micronuclei. Folic acid supplementation was found to revert the effects of oxidative stress in diabetic patients [57]. Additionally, folic acid supplementation has been shown to reduce glycosylated hemoglobin (HbA1C), fasting blood glucose, insulin resistance, and homocystinuria in T2DM [58].

One meta-analysis showed that folic acid supplementation was associated with lower tHcy concentrations, compared to placebo, in patients with T2DM (WMD: −3.52 μmol/L; 95%CI: −4.40, −2.6; *p* < 0.001), but not HbA1c (I2 = 43.7%; *p* = 0.149) [59]. Another study found that high RBC folate was a significant predictor of insulin resistance and other metabolic complications in obese patients [33]. A dose-response meta-analysis of RCTs showed that folic acid supplementation significantly reduced fasting blood glucose (WMD: −2.17 mg/dL; 95%CI: −3.69, −0.65; *p* = 0.005) and Homeostatic Model Assessment for Insulin Resistance (HOMA-IR) (WMD: −0.4; 95%CI: −0.70, −0.09; *p* = 0.011). However, these reductions were considered small, and their clinical implications may not be significant. In the same analysis, investigators found no effect of folic acid supplementation on HbA1C concentrations [40]. In another meta-analysis of RCTs, Lind et al. [60] found that folic acid supplementation, compared to placebo, reduced fasting insulin and HOMA-IR, but did not affect the fasting glucose and HbA1c. Based on the evidence, the association between folic acid supplementation and markers of glucose regulation is somewhat inconsistent. To date, no studies have been conducted on the effect of folic acid fortification on markers of glucose regulation. Therefore, further studies are needed to determine whether mandated folic acid fortification has any favorable impact on glucose regulation in healthy individuals and those with diabetes.

Emerging evidence suggests that the gut microbiome may play a role in the development of T2DM [61]. An imbalance in the gut microbiome can lead to inflammation and metabolic dysfunction, contributing to the development of insulin resistance and subsequent development of T2DM [62]. Potential mechanisms include alterations in gut permeability, production of microbial metabolites, altered lipid metabolism, and modulation of host gene expression [63]. On the other hand, probiotic bacteria such as *Bifidobacterium* and *Lactobacillus* have been studied as a source of folic acid for the host [64]. Vitamins such as folic acid from the unabsorbed diet can serve as a growth factor for colonic bacteria [65,66]. It has been suggested that folate may help to promote the growth of beneficial gut bacteria while suppressing harmful ones, regulating the host’s immune cell function, and improving drug efficacy [66]. While the relationship between folate fortification, the gut microbiome, and diabetes is still being explored, some studies have suggested that increasing folate intake may have a positive impact on glycemic control in individuals with diabetes [40]. However, more research is needed to fully understand the mechanisms underlying this three-way intricate relationship between microbiome, diabetes, and folic acid fortification.

## 4. Conclusions

The current implementation of mandatory folic acid fortification has achieved its intended benefit of reducing the incidence of NTDs in newborns. Along with this, there have been a few unintended benefits that have improved the health status of some populations, including a reduction in tHcy concentrations, which is considered an independent risk factor for CVD. Other unintended benefits of folic acid fortification include virtually eliminating folic acid deficiency from the US, reducing the prevalence of anemia, and decreasing hemoglobin concentrations. However, the effect of folic acid fortification on cognitive health, stroke, and diabetes is not very clear. Further research is needed to clarify these unintended benefits.

While mandated folic acid fortification has been effective in reducing the incidence of NTDs, there are also some concerns associated with mandatory fortification. Some of the concerns are as follows:

- Increased intake of folic acid: excessive consumption of folic acid can mask the symptoms of vitamin B-12 deficiency, which can lead to vitamin B-12-induced anemia and neurological damage due to possible delayed diagnosis.
- Increased cancer risk: some studies have suggested that high folic acid intakes may increase the risk of certain types of cancer. However, the evidence for this is not very conclusive.
- Unmetabolized folic acid (UMFA) concentrations: there are some concerns that UMFA may have adverse effects on health, although the evidence for this is not very conclusive yet.
- Fortification of unhealthy foods: mandatory folic acid fortification may lead to the fortification of unhealthy foods or the displacement of other essential nutrients.
- Individual choice: some individuals may choose to avoid foods that have been fortified with folic acid due to personal beliefs or dietary restrictions.
- Socioeconomic disparities: Fortification may not be accessible to all segments of the population leading to socioeconomic disparities in folic acid intakes.

It is important to note that the benefits of folic acid fortification in reducing the incidence of NTDs outweigh the potential risks associated with its mandatory folic acid fortification. However, the long-term effects of folic acid fortification need to be evaluated in non-target populations such as the elderly, children, men, post-menopausal women, and patients who are on antimetabolites for cancer treatment. Specifically, the elderly tend to consume more supplements, and children tend to consume more breakfast cereals, which are known to contain high levels of folic acid. These two populations should be monitored more closely for any long-term health effects.

## Figures and Tables

**Table 1:** Summary of studies that assessed health benefits of folic acid fortification ^1^.

Reference (Author/s and Year)	Study Design	Intervention/Data Collection	Outcome Measurements	Findings	Conclusions
Intended benefits of folic acid fortification					
Bol et al., 2006 [22]	Retrospective cohort	Between 1995 to 2001	Spina bifida and encephalocele	↑Dietary Folic acid, ↑First-year survival rate; no difference in encephalocele	In addition to preventing the occurrence of NTDs, folic acid may have a role in a role in reducing the severity of NTDs
Ho et al., 2021 [23]	Systematic review	Evidence searched from 1 January 1990 to 31 August 2020	Spina bifida and infant mortality rate	↓spina bifida and associated infant and neonatal mortality rates	Significant declines in spina bifida associated infant/neonatal mortality and case fatality. Likely due to folic acid fortification
Unintended benefits of folic acid fortification					
Ganji et al., 2006 [26]	Retrospective cohort	From 1988 to 2002 pre- and post-fortification	Serum folic acid, RBC, RBC folate, and tHcy concertation	↑Dietary folic acid, ↓tHcy	Folic acid plays a role in the reduction in tHcy.
Holmes et al., 2011 [27]	Meta-analysis	Mean follow-duration of 4.7 y	Risk of stroke; effect modification by population	↑OR stroke was higher in Asia than in America, Australia, and New Zealand	Stroke risk in Asia was higher in comparison to areas with folate fortification such as America, Australia, and New Zealand.
Yang et al., 2006 [28]	Cohort study	From 1990–2002	Stroke mortality	↓Stroke mortality with folate fortification	Stroke mortality decreased after mandatory folic acid fortification.
Morris et al., 2007 [29]	Cohort study	From 1999–2002; folic acid fortification in elderly ˃60 y of age	Cognitive impairment	↑High serum folate; ↓B12 deficiency; ↑anemia and cognition; Normal B12 ↑serum folate, ↓cognitive impairment	Folic acid has two-sided effects on cognitive health depending on the serum B12 serum concentration.
Biemi et al., 2021 [30]	Retrospective, observational	2004–2010 pre- and post-fortification in children 5–14 y of age	Hemoglobin, hematocrit, RBC, MCV, and anemia	No difference in mean hemoglobin; ↑MCV concentrations post-fortification	No change in anemia in post-fortification; however, MCV significantly increased suggesting an increase in B12 deficiency.
Ganji et al., 2009 [31]	Cohort study	1988–2004, pre and post-folic acid fortification	Anemia and macrocytosis	↑Dietary folic acid; ↓anemia in women	Improvement in hemoglobin and decreased prevalence of anemia after folic acid fortification.
Carrasco Quintero et al., 2013 [32]	Randomized control trial	In 2010, maize flour fortified with folic acid	Hemoglobin in women	↑Hemoglobin	Fortified flour is a good option for regionalized women in rural areas who are underweight, undernourished, and have anemia.
Li et al., 2018 [33]	Cross-sectional study	2011–2012, serum folic acid	Serum folate and insulin resistance (fasting plasma glucose, OGTT, serum insulin, and HOMA-IR)	↑Serum folate; ↓HOMA-IR	Serum folate was inversely associated with insulin resistance

^1^ Abbreviations: B12, vitamin B-12; HOMA-IR, homeostatic-model assessment for insulin resistance; MCV, mean corpuscular volume; NTD, neural tube defect; OGTT, oral glucose tolerance test; OR, odds ratio; RBC, red blood cells; RCT, randomized controlled trials; tHcy, total homocysteine.
